# Effects of psycho-educational intervention on health-related quality of life (QOL) of patients with chronic liver disease referring to Shiraz University of Medical Sciences

**DOI:** 10.1186/1477-7525-3-81

**Published:** 2005-12-16

**Authors:** Farkhondeh Sharif, Sadrollah Mohebbi, Hamid-Reza Tabatabaee, Mehdi Saberi-Firoozi, Sakineh Gholamzadeh

**Affiliations:** 1 Psychiatric Nursing Department, Shiraz University of Medical Sciences, Shiraz, Iran; 2 Psychiatric Nursing Department, Shiraz University of Medical Sciences, Shiraz, Iran; 3 Gastroenterology Department and Liver Research Center, Shiraz University of Medical Sciences, Shiraz, Iran; 4 Medical-Surgical Nursing Department, Shiraz University of Medical Sciences, Shiraz, Iran; 5 Biostatistics Department, Shiraz University of Medical Sciences, Shiraz, Iran

## Abstract

**Background:**

Chronic liver diseases (CLDs) are progressive disorder which has a significant impact on the well-being of patients and leads to significant morbidity. CLDs are characterized by disturbances in physical, psychological and social aspects of well-being. It causes significant health-related quality of life (QOL) impairment. Psycho-educational interventions targeting to functional factors could be beneficial for patients with CLDs.

**Methods:**

An interventional study was conducted on 110 patients with CLDs in Shiraz Liver Transplantation Center (SLTC). Subjects with the required CLDs criteria were selected and randomly divided into experimental (55) and control (55) groups. A two part questionnaire with 25 items concerning demographic and general information and 29 items regarding QOL was used. The psycho-educational needs of the experimental group were assessed in a session before the intervention, then the experimental group took part in 3 sessions individually and one session in groups. The questionnaires were filled in again for both groups but the control group did not receive the intervention program. The questionnaires were filled in again for both groups one day and three months after the intervention.

**Results:**

Findings revealed no significant differences between the two groups from the view point of demographic characteristics such as marital status, gender, etc... (p > 0.05) and from the point of clinical variables no statistically significant differences were found (p > 0.05).

Findings revealed statistically significant differences in all domain of QOL in the experimental group three months after the intervention (p = 0.001), while there was no statistically significant differences in the control group.

**Conclusion:**

The results of this study revealed that psycho-educational intervention had a significant effect on QOL of patients with CLDs.

## Background

Chronic liver diseases (CLDs) are an extensive disorder affecting 5 million people in the United States and are one of the leading causes of death [1]. People with chronic HBV infection are at a lifetime risk of developing hepatocellular carcinoma (HCC) or cirrhosis, or both. Many people with HBV are unaware that they are bearer the infection, while of those who are chronically infected, only a minority receive routine, scheduled follow-up to monitor their disease status. [2-4]. Chronic hepatitis C virus (CHCV) infection is a prevalent and extensive condition leading to cirrhosis in up to 20% of those chronically affected [1,2]. Patient with CLDs suffer from debilitating fatigue, pruritus, loss of esteem, depression, and complications of cirrhosis [1,5]. CLDs lead to reduction of health-related quality of life (QOL) as reflected in the disturbances of cognitive, behavioral, physical and social aspects of well-being and therefore lead to physical and psychological problems [6]. The impact of chronic hepatitis on QOL and creating complications such as encephalopathy, variceal hemorrhage and ascitis is well recognized [7].

A large number of studies have been performed regarding QOL of patients with CLDs in terms of assessing the impact of treatment intervention however, there is little information concerning QOL for such patients and there is relatively little published evidence concerning the effectiveness of psycho-educational intervention. According to our knowledge, the only published related article regarding QOL of patients suffering from CLDs in Iran has been conducted on the effect s of self-care program on QOL of 44 patients referring to Tehran Hepatitis Center. The self care questionnaire and CLDQ for measuring the QOL was used. Then self-care educational program was conducted and the patients were followed for 3 months. The results confirmed the positive effects of the educational and self care program on the QOL of cirrhotic patients. The QOL significantly improved in the experimental group after the intervention(p= 0.001), while the QOL decreased in the control group [9]. The objective of this study was to investigate the effect of psycho-educational intervention on QOL of patients with CLDs referring to the SLTC.

## Methods

In this study a randomized controlled clinical trial was used. The study was consisted two groups of subjects. One hundred and ten patients with CLDs out of 1585 patients who were registered by the Shiraz University of Medical Sciences Liver Transplantation Center were selected according to inclusion criteria. They were randomly assigned into experimental (55) and control (55) groups. The diagnosis of both groups and blood tests was approved by gastroenterologist. Patients were 18 years and older, not having other chronic illness such as malignancy, diabetes, mental illness, preferably living in Shiraz and close cities and patients with chronic hepatitis c were in the expected list of liver transplantation. The patients were registered at the liver transplantation center or liver clinic. The SLTC is covering all the chronic liver disease patients in Fars province. At the time of study from 1585 registered cases, 1128 of them had been chronic hepatitis B and 457 of cases chronic hepatitis C which 191 of them liver transplanted and 337 child score of C in transplant waiting list.

### Data collection tools

In this study two part questionnaire and one "needs assessment" session were used. The first part of questionnaire was consisted of 23 items demographic and general information questions. It included age, gender, education level, marital status, number of admission, number of children, the duration of illness, financial status, number of admission in hospital, the cause of disease, kind of medication that they were taking and complication. The second part of questionnaire was about CLDs QOL which is the first liver specific instrument for measuring QOL in chronic liver disease developed by Younossi et al.[8]. It includes 29 items in the 6 domains which are abdominal symptoms, fatigue, activity, worry, emotional functions and systemic symptoms. It is 7 point likert scale type of answers ranging from "all of the time" to "none of the time". The main advantage of CLDs QOL scale is its wide application in hematologic studies. The construct validity of the scale has been supported with a strong correlation with patients' global rating scores (r = 0.84; p = 0.02). The fatigue domain of CLDQ, both correlated highly with GRC (r = 0.83 and r = 0.90, respectively; p = 0.006) [7,8].

Also the content validity of translated CLDs QOL was approved by all faculty members of Gastroenterology Department. Reliability was determined from test-retest on 30 patients with the interval period of 10 days for test-retest. Spearman's rank correlation coefficient was r = 0.98 (p < 0.001).

At the beginning in the experimental group patients were asked to attend one discussion session to assess their needs. Their needs were knowledge about the nature of disease, sign and symptoms of disease, physical limitation, nutrition, adaptation and adjustment to chronic illness, social function, anxiety and drug therapy.

Both groups filled out the questionnaires, then the experimental group was divided into 5 subgroup, according to their disease stage, level of education and their free time. Each group consisting of 11 patients, and four interventional program sessions (three sessions individually in 3 weeks and one session in group) were conducted only for the experimental group. Each session lasted for 90 minutes and one of the close patients' relative was attended in individual session.. When family members have enough information, they play a supportive and helpful role to maintain clients' need... The psycho-educational intervention program consisted information relating to CLDs and its effect on QOL, adjustment to chronic disease (coping strategies), relaxation, exercise, diet and nutrition, drugs used and possible side effects. Posters and handouts were used as training aids in each session based on the subjects' educational level... Educational booklet was given to all patients. The questionnaires were filled in again by both groups one day and three months after the intervention... After data collection, the educational booklet was given to the control group to provide equity too. Data were controlled, encoded and analyzed after data collection.

### Ethical consideration

The study was conducted after approval had been obtained from Vice-Chancellor for research of Shiraz University of Medical Sciences.. All participants were informed of the objective and design of the study and written consents were received from the participants for interview.

## Results

The majority of subjects were men (76.4% in the experimental group and 74.5% in the control group). From the point of marital status 74.5% in the experimental and 67.3% in the control group were married. Findings revealed no significant differences from view point of gender and marital status between two groups (P > 0.05), Table 1.

**Table 1:** Frequency distribution of demographic and clinical characteristics of patients in the control and experimental group

	**Experimental**		**Control**		
**Items**	n	**%**	n	**%**	**P**
**Gender**					
Male	42	76.4	41	74.5	> 0.05
Female	13	23.6	14	25.5	
**Marital Status**					
Married	41	74.5	37	67.3	> 0.05
Single	14	25.5	16	29.1	
**Education Level**					
illiterate	4	7.3	6	10.9	= 0.29
Primary school	10	18.2	17	30.9	
Secondary school	15	27.3	15	27.3	
Diploma and higher	26	47.2	17	31	
**Etiologic factor**					
Hepatitis C	23	41.8	20	36.4	= 0.99
Hepatitis B	30	54.5	32	58.2	
Other cause	2	3.6	3	5.4	
**Duration of disease**					
6 month to one year	14	25.5	14	25.5	= 1.0
More than 1 to 2 year	13	23.6	13	23.5	
More than 2 year	28	50.9	28	50.9	
**No of Admission**					
Once	5	9.1	2	3.6	= 0.32
Twice	2	3.6	2	3.6	
Triple and more	2	3.6	--	--	
No admission	46	83.6	51	92.7	
**Total**	55	100	55	100	

Chronic hepatitis B has been diagnosed in 54.5% of the cases in the experimental group and 58.2% of the cases in the control group. Duration of disease in both groups has been more than two years. Using of Wilcoxon signed ranks test, findings revealed in patients of experimental group in four domains of QOL, such as fatigue, emotional function, worry and systemic symptoms, one day after the intervention a significant difference was shown (p < 0.001) whereas, in two domains, such as activity and abdominal symptoms, no significant difference was observed. Three months after intervention, statistically significant differences in all domains of QOL were shown (p < 0.001). Table 2.

**Table 2:** Comparison of Mean score of CLDQ domains in the experimental group (pre and post test no 1 and 2

	**Pre-test**		**Post test 1**			**Post test 2**		
**Variables**	Mean	SD	Mean	SD	P value	Mean	SD	P value
Abdominal symptoms	7.12	4.60	7.40	4.49	0.90	5.25	2.76	0.001*
Fatigue	19.34	6.60	17.98	6.34	0.001	12.18	5.11	0.001*
Activity	9.58	3.88	9.34	3.48	0.369	7	2.91	0.001*
Emotional Function	36.40	8.07	34.54	7.80	0.001	20.10	7.26	0.001*
Worry	27.63	4.59	25.20	4.53	0.001	13.65	4.81	0.001*
Systemic symptoms	19.33	6.61	17.97	6.33	0.001	12.17	5.12	0.001*
Total score	90.50	19.19	85.12	18.93	0.001	51.21	17.40	0.001*
Total	55		55			55		

A significant difference in three domains of QOL, such as abdominal symptoms, emotional function and worry was seen in control group one day and three months after intervention, but in three domain of QOL, such as fatigue, activity and systemic symptoms, no statistically significant differences were observed after one day and three months after intervention using Wilcoxon test (Table 3). Mean comparison of different QOL domains in both experimental and control groups before intervention and one day after intervention showed statistically significant differences in two domains of QOL such as fatigue and emotional function between groups, but three months after intervention showed statistically significant differences in all domains of QOL in the experimental group using Mann-Whitney U test (p < 0.001, Table 4). So, the study hypothesis of psycho-educational intervention is effective on QOL of patients with CLDs was approved.

**Table 3:** Comparison of Mean score of CLDQ domains in the control group (pre and post test no 1 and 2)

	**Pre-test**		**Post test 1**			**Post test 2**		
**Variables**	Mean	SD	Mean	SD	P value	Mean	SD	P value
Abdominal symptoms	7.58	3.63	7.98	3.64	0.001*	8.23	3.28	0.001*
Fatigue	18.87	6.68	18.87	6.13	0.667	18.34	5.63	0.117
Activity	9.05	3.90	8.85	3.88	0.133	9.147	3.56	0.629
Emotional Function	35.85	8.48	35.36	8.53	0.021*	34.47	8.48	0.001*
Worry	26.78	4.43	25.32	4.26	0.001*	24.38	4.44	0.001*
Systemic symptoms	18.86	6.67	18.85	6.12	0.668	18.33	5.62	0.116
Total score	90.50	19.19	85.12	18.93	0.001*	51.21	17.40	0.001*
Total	55		55			55		

**Table 4:** Comparison of mean differences score of CLDQ domains in the control and experimental groups

	**Differences of pre-test score and first follow-up**				
	**Experimental group**		**Control group**		
**Variables**	Mean	SD	Mean	SD	P value
Abdominal symptoms	- 0.27	1.33	- 0.40	0.82	0.364
Fatigue	1.36	1.96	0.001	1.57	0.001*
Activity	0.23	1.57	0.20	0.95	0.898
Emotional Function	1.85	4.16	0.49	1.76	0.024*
Worry	2.43	2.89	1.45	1.56	0.082
Systemic symptoms	2.42	2.88	1.43	1.55	0.081
Total score	5.38	8.54	1.54	2.67	0.001*
	**Differences of pre-test score and second follow-up**				
	**Experimental group**		**Control group**		
	Mean	SD	Mean	SD	P value

Abdominal symptoms	1.87	12.64	-0.65	1.32	0.001*
Fatigue	7.16	3.35	0.52	2.61	0.001*
Activity	2.58	2.24	0.09	1.63	0.001*
Emotional Function	16.29	6.19	1.38	2.66	0.001*
Worry	13.98	4.24	2.40	2.60	0.001*
Systemic symptoms	7.14	3.33	0.51	2.60	0.001*
Total score	39.30	12.73	3.65	5.44	0.001*

## Discussion

Findings revealed that, intervention was effective on all aspects of QOL after 3 months in the experimental group. The inability of the intervention program to provide evidence of significant changes on all aspects of QOL from pre to one day after the intervention may be due to the various reasons. One may be that, four sessions may not have provided sufficient time for patients to effectively learn and develop the necessary skills regarding QOL. Besides receiving knowledge and information about disease to keep their good QOL and relief their anxiety, long term behavioral and attitude changes need to take place which can occur only over a longer period of time.

In general, the result of this study revealed that psycho-educational intervention had an improving effect on QOL of patients with CLDs. This result is supported by a study indicating the beneficial effects of training and self-care programs on the health related QOL of Iranian patients with CLDs [9,10]. According to Kato and Ishii, training session on liver disease and interchange of information among patients at group work is helpful to reduce their anxiety related to their disease [11].

The result of this study showed that the majority of patients in experimental (76.4%) and control group (74.5%) were male. These findings are supported by the previous studies conducted by Gledhill, et al. and Alavian, et al. and Zandi et al. [12,13,9].

The finding of this study revealed that none of the patient in both group had any other diseases and no significant differences between two groups were found (P = 0.69). This findings is in contrast to the finding of Khozema, et al. [14] who found out that 71% of their patients had history of other diseases and it was statistically significant (p < 0.001).

The findings of this study showed that most of the patients in the experimental group 54.5% and in the control group 58.2% had hepatitis B but not statistically significant differences were showed (P = 0.47). This finding is similar to the study conducted by Burke et al. [15]. The higher rate of Hepatitis B may be due to many reasons. The findings revealed that the majority of patients in experimental (76.4%) and control group (74.5%) were male. Male are more susceptible to high risk behavior, using harmful drugs, alcohol and engaging in sexual activity without protection.

The result of the present study have added support to an earlier study by Hauser, et al.(2004) who found out that health related QOL in chronic hepatitis C is not determined by severity of liver diseases but psychiatric and medical co-morbidities and disease-related worries [16].

## Conclusion

The results of need assessment of patients with CLDs supported a need for an intervention program to increase their QOL.

The findings revealed that there was a significant difference in three domain of QOL, such as fatigue, emotional function, worry and systemic symptoms for patients in the experimental group one day after the intervention, whereas, A statistically significant difference in all domains of QOL was shown in three months after the intervention. A statistically significant difference in three domains of QOL, such as abdominal symptoms, emotional function and worry was observed in control group one day and three months after intervention, but in two domains of QOL, such as fatigue and activity, no statistically significant difference was seen after one day and three months after intervention.

Generally, the result of this study revealed that psycho-educational intervention had an improving effect on QOL of patients with chronic liver diseases. It seems that the combination of psychological and educational intervention leads to improvement of QOL.

## Authors' contributions

FSH: Initiation and coordination of the study, editorial and revision of draft papers, translation of draft papers and writing the manuscript, SM: Data collection and writing the first draft. MSF: advisor, SGH: advisor, HRT: statistical advisor.

**Table 5:** Structured format of psycho-educational intervention

**Session**	**Topic of discussion**
1	Introduction to liver and its function, sign and symptoms of chronic liver diseases
2	Treatment and Medication and possible side effects, adjustment to chronic disease, coping strategy
3	Exercise, relaxation exercise, anxiety reduction, diet and nutrition
4	Open group and open discussion
